# Effects of volume-targeted pressure-controlled inverse ratio ventilation on functional residual capacity and dead space in obese patients undergoing robot-assisted laparoscopic radical prostatectomy

**DOI:** 10.1016/j.bjao.2022.100020

**Published:** 2022-07-20

**Authors:** Go Hirabayashi, Yuuki Yokose, Hiroyuki Oshika, Minami Saito, Koichi Maruyama, Tomio Andoh

**Affiliations:** Department of Anaesthesiology, Mizonokuchi Hospital Teikyo University School of Medicine, Kanagawa, Japan

**Keywords:** dead space, functional residual capacity, pressure-controlled inverse ratio ventilation, robot-assisted laparoscopic radical prostatectomy, volumetric capnography

## Abstract

**Background:**

The effect of inverse inspiration:expiration (I:E) ratio on functional residual capacity (FRC) during pneumoperitoneum is unclear. We hypothesised that volume-targeted pressure-controlled inverse ratio ventilation (vtPC-IRV) would increase FRC by increasing the level of auto-PEEP in low respiratory compliance situations.

**Methods:**

During robot-assisted laparoscopic radical prostatectomy, 20 obese patients were sequentially ventilated with four different settings for 30 min in each setting: (1) control, I:E ratio of 1:2 and baseline airway pressure (BAP) of 5 cm H_2_O; (2) IRV2, I:E ratio of 2:1 and BAP off; (3) IRV3, I:E ratio of 3:1 and BAP off; and (4) IRV4, I:E ratio of 4:1 and BAP off. The changes in FRC were identified and compared among these settings.

**Results:**

The FRC significantly increased as the I:E ratio increased. The FRC values expressed as median (inter-quartile range) during control, IRV2, IRV3, and IRV4 were 1149 (898–1386), 1485 (1018–1717), 1602 (1209–1775), and 1757 (1337–1955) ml, respectively. Auto-PEEP increased significantly as the I:E ratio increased and correlated with FRC (rho=0.303; *P*=0.006). Shunt and physiological dead space were significantly lower in all IRV groups than in the control group; however, there were no significant differences among the IRV groups.

**Conclusions:**

vtPC-IRV with shortened expiratory time and increased auto-PEEP effectively increases FRC during robot-assisted laparoscopic radical prostatectomy in obese patients. FRC increases progressively as the I:E ratio increases from 1:2 to 4:1; however, an I:E ratio higher than 2:1 does not further improve the dead space.

**Clinical trial registration:**

UMIN000038989.

Carbon dioxide pneumoperitoneum in a steep Trendelenburg position induces atelectasis and reduces the functional residual capacity (FRC).^1^^,^^2^ Obese patients are at a higher risk of atelectasis with reduced FRC and exhibit worse respiratory mechanics than non-obese patients.^3^, ^4^, ^5^, ^6^, ^7^, ^8^, ^9^

We previously reported that the physiological dead space (VD_phys_) in robot-assisted laparoscopic radical prostatectomy improved with volume-targeted pressure-controlled inverse-ratio ventilation (vtPC-IRV).^10^, ^11^, ^12^ vtPC-IRV with a prolonged plateau time promotes sufficient opening of the slow-opening alveoli and facilitates diffusion of gases from the pulmonary capillaries to the alveoli, resulting in reduced VD_phys_. However, VD_phys_ increases with time, suggesting that atelectasis develops with time during surgery. FRC, auto-PEEP, or total PEEP were not measured in those studies. No previous studies have investigated the effect of inverse inspiration:expiration (I:E) ratio with short expiratory time on FRC during pneumoperitoneum in the Trendelenburg position. Thus, we aimed to evaluate the FRC with various I:E ratios of vtPC-IRV in obese patients undergoing robot-assisted laparoscopic radical prostatectomy.

We hypothesised that vtPC-IRV with a short expiratory time would increase FRC by increasing the level of auto-PEEP. Our study contributes to the practice of open-lung ventilation in situations with low static compliance.

## Methods

### Study design and patients

This single-centre prospective interventional study was conducted at the Mizonokuchi Hospital, Teikyo University School of Medicine, Kanagawa, Japan, and was approved by the Ethics Committee of the Teikyo University School of Medicine, Tokyo, Japan (chairperson and dean: M. Kawamura) on 15 April 2019 (No. 18–194). It was registered with the University Hospital Medical Information Network Clinical Trials Registry (UMIN000038989). Written informed consent was obtained from all participants.

The study included patients with BMI >25 kg m^−2^ aged 40–75 yr with ASA physical status score 1–2, scheduled to undergo robot-assisted laparoscopic radical prostatectomy. The exclusion criteria were ASA physical status of 3–5, a history of asthma, pulmonary emphysema, pneumothorax, or lung surgery. The patients were enrolled between 15 June 2019 and 30 July 2020.

### Anaesthesia protocol

Routine patient monitoring included ECG, pulse oximetry, noninvasive arterial blood pressure measurement, and inspired and expired CO_2_ analysis. Continuous radial arterial pressure, cardiac index, and stroke volume variation (SVV) were monitored using a Vigileo system with a Flo-Trac sensor (Edwards Lifesciences, Irvine, CA, USA). Mainstream CO_2_ and flow sensors were attached to the proximal end of the tracheal tube to enable volumetric capnography (Senko Medical Instrument Co. Ltd., Tokyo, Japan). Anaesthesia was induced by administering i.v. propofol 1–3 mg kg^−1^ and fentanyl 2–4 μg kg^−1^. Tracheal intubation was performed after the administration of rocuronium 0.8–1.0 mg kg^−1^. Anaesthesia was maintained by total intravenous administration of propofol 100–200 μg kg^−1^ min^−1^ and remifentanil 0.2–0.3 μg kg^−1^ min^−1^. Intermittent intravenous injections of rocuronium 0.1–0.2 mg kg^−1^ and fentanyl 1–2 μg kg^−1^ were administered, as needed.

### Interventions and ventilatory settings

After the induction of anaesthesia, we ventilated the patients' lungs using a ventilator (Engström Carestation™; Datex-Ohmeda, GE Healthcare, Helsinki, Finland) that measures FRC and auto-PEEP. After positioning in the 25–30° Trendelenburg position and CO_2_ pneumoperitoneum at 12 mm Hg, the patients' lungs were sequentially ventilated using four different settings for 30 min each: control, IRV2, IRV3, and IRV4 settings. Each ventilator setting included the volume targeted pressure control (expressed as ‘pressure control ventilation-volume guarantee’ in Engström Carestation™) mode, in which the airway pressure was adjusted to achieve a target tidal volume. The target tidal volume was adjusted with the plateau pressures permitted to increase to an upper limit of 30 cm H_2_O. The initial fraction of inspiratory oxygen (F_i_O_2_) was 0.5, and the ventilatory frequency was set at 12 bpm. The target tidal volume and ventilatory frequency remained constant throughout the study providing there were no respiratory complications. For the control setting, the I:E ratio was 1:2, and the baseline airway pressure (BAP; baseline at a set airway-pressure level, replacing a PEEP setting^13^) was 5 cm H_2_O. In IRV2, the I:E ratio was 2:1 and BAP was 0 cm H_2_O (off). In IRV3, the I:E ratio was 3:1 and BAP was off. In IRV4, the I:E ratio was 4:1 and BAP was off.

Auto-PEEP was allowed to increase to 10 cm H_2_O, peripheral oxygen saturation was allowed to drop to 93%, whereas end-tidal carbon dioxide (E_T_CO_2_) was permitted to increase to 60 mm Hg. When any of these predetermined limits were exceeded, the study was halted, and the ventilator setting was changed to an I:E ratio of 1:1, increasing the ventilatory frequency and F_i_O_2_ and increasing or decreasing the tidal volume.

The MAP was maintained >70 mm Hg using IV ephedrine (4–8 mg). An intravenous fluid challenge was provided with 10 ml kg^−1^ of Ringer's acetate solution or hydroxyethyl starch if the SVV exceeded 15%.

### Outcome measures

The primary outcome measure was FRC measured using the oxygen wash-in and wash-out methods provided within the ventilator (Engström Carestation™). The International Organization for Standardization (ISO) redefined the baseline and PEEP terminology.^13^ PEEP is the actual and measured value of respiratory pressure at the end of an expiratory phase. BAP is the quantity by which the BAP positively offsets from the ambient pressure, replacing a PEEP setting. Auto-PEEP is the portion of the stabilised airway pressure above that set for the end-expiratory pressure, at the end of an expiratory-hold procedure that temporarily occludes the airway in the absence of any respiratory activity. Total PEEP is the stabilised airway pressure at the end of an expiratory-hold procedure that temporarily occludes the airway in the absence of any respiratory activity. The ventilator measured auto-PEEP (expressed as ‘PEEP*i*’ in Engström Carestation™) and total PEEP were calculated as follows: total PEEP=auto-PEEP+PEEP. Static compliance (C_stat_) was calculated as C_stat_=V_TI_ (P_plat_–total PEEP)^−1^, where V_TI_ is the inspired tidal volume and P_plat_ is the plateau pressure.

**Fig. 1:**
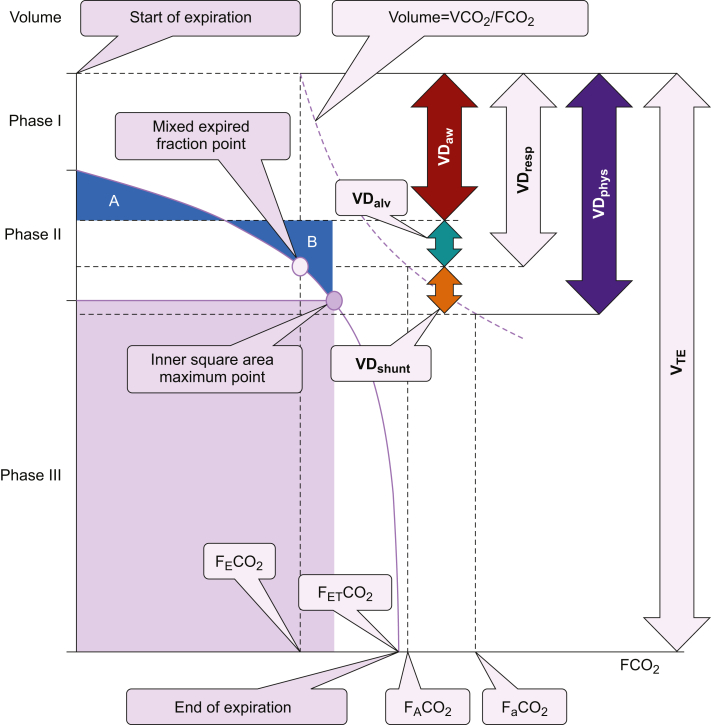
Novel theory of volumetric capnography. The expired tidal volume on the Y axis is plotted against the partial pressure of expired CO_2_ on the X axis to express the curve of ‘Volume=VCO_2_/FCO_2_’. Phase I represents CO_2_-free and pure dead space, phase II represents the transition between the airway and alveolar gas, and phase III represents the alveolar gas. Phase II ends at the inner square area maximum point. VD_aw_ is determined by applying Fowler's equal area method (area A equals to B). The volume from the start of expiration to the partial pressure of the mixed expired CO_2_ point on the Y axis is defined as respiratory dead space (VD_resp_). Thus, alveolar dead space (VD_alv_) is calculated as VD_resp_–VD_aw_ and shunt dead space (VD_shunt_) is calculated as VD_phys_–VD_resp_. VD_phys_, physiological dead space; VD_aw_, airway dead space; VD_alv_, alveolar dead space; VD_shunt_, shunt dead space; V_TE_, expired tidal volume; VCO_2_, expired tidal volume of CO_2_; FCO_2_, fractional concentration of CO_2_ [FCO_2_=*P*co_2_ (P_B_–P_H2O_)^−1^=Pco_2_ (760–47)^−1^; P_B_, barometric pressure; P_H2O_, water vapor pressure at 37°C]; F_E_CO_2_, mixed expired FCO_2_; F_ET_CO_2_, end-tidal FCO_2_; F_A_CO_2_, alveolar FCO_2_; and FaCO_2_, arterial FCO_2_.

Physiological dead space (VD_phys_) is a functional evaluation of the difference in the partial pressure of CO_2_ between the arterial blood and mixed expired gas [VD_phys_=V_TE_･(*P*aco_2_–*P*_E_co_2_)･*P*aco_2_^−1^], where V_TE_ is the expired tidal volume and *P*eco_2_ is the mixed expired gas partial pressure of CO_2_, representing the overall CO_2_ elimination efficiency of the respiratory and circulatory dynamics. Using the volumetric capnography theory (see Supplementary data), VD_phys_ was divided into airway dead space (VD_aw_), alveolar dead space (VD_alv_), and shunt dead space (VD_shunt_) (VD_phys_=VD_aw_+VD_alv_+VD_shunt_) (Fig 1). VD_aw_ is a functional evaluation of the airway space volume, including the gas diffusion effect, representing extra-alveolar V̇_A_/Q̇=∞ mismatch. Respiratory dead space (VD_resp_) is a functional evaluation of the difference in CO_2_ partial pressure between the alveolar and mixed expired gas, representing V̇_A_/Q̇>1 mismatch. VD_alv_, calculated as VD_resp_–VD_aw_, is a functional evaluation of the relative hyperinflation in the alveolar units, representing intra-alveolar V̇_A_/Q̇>1 mismatch. VD_shunt_, calculated as VD_phys_–VD_resp_ or VD_phys_–VD_aw_–VD_alv_, represented functional evaluation of relative hyperperfusion and the difference in CO_2_ partial pressure between the pulmonary artery and mixed expired gas, representing V̇_A_/Q̇<1 mismatch.

Each component had two different influential factors: the plateau-dependent dead space change caused by different ventilator settings and the plateau-independent dead space change caused by the time elapsed under the same ventilator settings. Plateau-independent VD_aw_ change represented extra-alveolar V̇_A_/Q̇=∞ mismatch derived from airway space volume change. Plateau-dependent VD_aw_ change represented extra-alveolar V̇_A_/Q̇=∞ mismatch caused by gas diffusion in the airway. Plateau-dependent VD_alv_ change represented intra-alveolar V̇_A_/Q̇>1 mismatch caused by hyperinflation with circulatory suppression. The plateau-independent VD_alv_ change represented intra-alveolar V̇_A_/Q̇=∞ mismatch such as pulmonary infarction. Plateau-dependent VD_shunt_ change represented intra-alveolar V̇_A_/Q̇<1 mismatch caused by heterogeneous expansion disorders. Plateau-independent VD_shunt_ change represented intra-alveolar V̇_A_/Q̇=0 mismatch caused by atelectasis and extra-alveolar V̇_A_/Q̇=0 mismatch caused by an extra-alveolar shunt (Table 1).

**Table 1:**
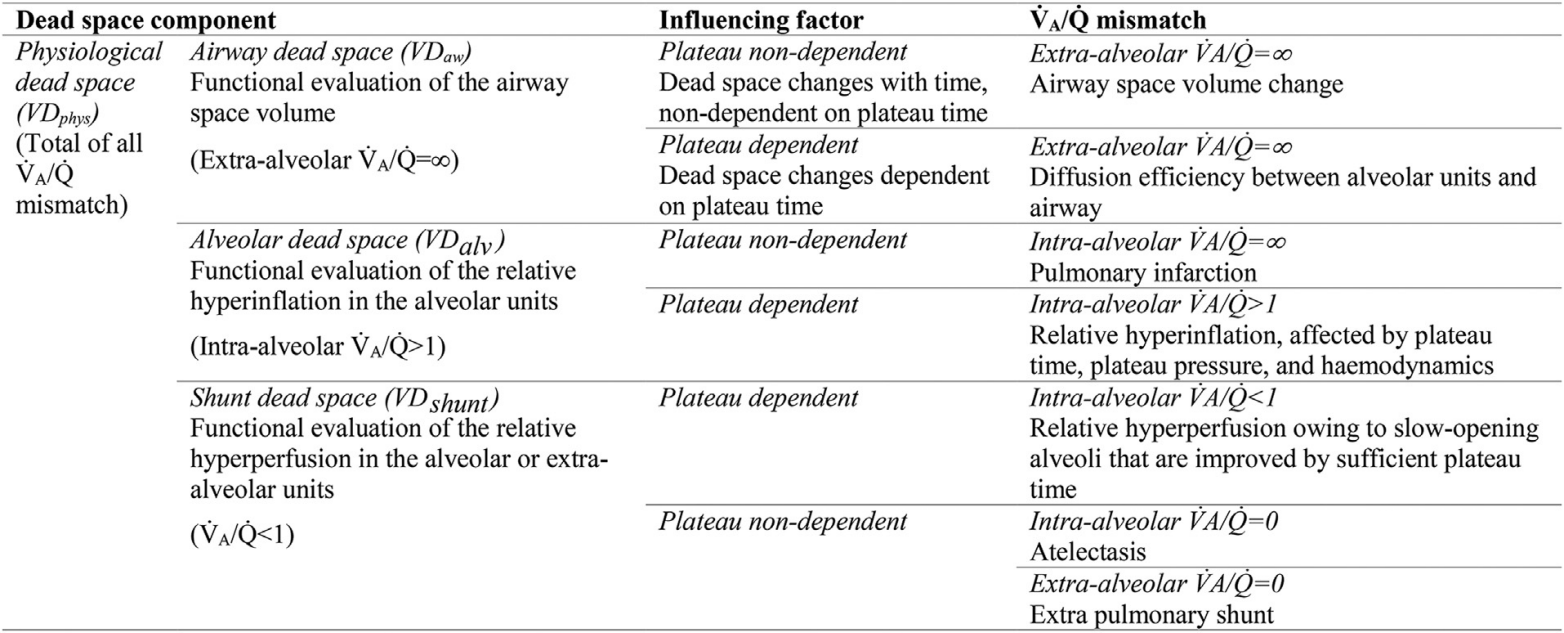
Interpretation of dead space components. V̇_A_/Q̇, ventilation to perfusion ratio.

In this study, we did not explore the mechanism by which the plateau-dependent or independent factors affected each component of the dead space because the dead space components were analysed only at the end of each ventilator setting for a 30-min period but not evaluated over time.

### Statistical analysis

The sample size was calculated based on previous data^14^^,^^15^ using paired samples *t*-test as 20 subjects per group to detect the differences in the FRC with a power of 0.8 and a type I error rate of 0.05, based on an estimated difference of 0.66 of the parameter's estimated standard deviation (sd). Non-parametric data distribution was assumed owing to the small number of patients included in this study, and the descriptive parameters were generally expressed as median (inter-quartile range [IQR]).

The Friedman test followed by the Wilcoxon signed-rank test with Bonferroni correction were used to identify the changes and differences in respiratory and haemodynamic variables among the ventilator settings. Multiple regression analysis was used to identify the influence of various factors on the primary outcome measure. The dependent variable was FRC, and the independent variables were selected from patient characteristics and ventilator settings based on clinical prediction. We eliminated the calculated or estimated variables from the potential independent factors. The selected independent variables were BMI, FVC (% predicted), forced expiratory volume in 1 s (FEV_1_, % predicted), target tidal volume, BAP, and I:E ratio. Spearman's rank correlation was used to assess the relationship between the variables. All statistical analyses were performed using R© version 3.5.2 (R Foundation for Statistical Computing, Vienna, Austria). Statistical significance was set at *P*<0.05.

## Results

### Patient characteristics

**Fig. 2:**
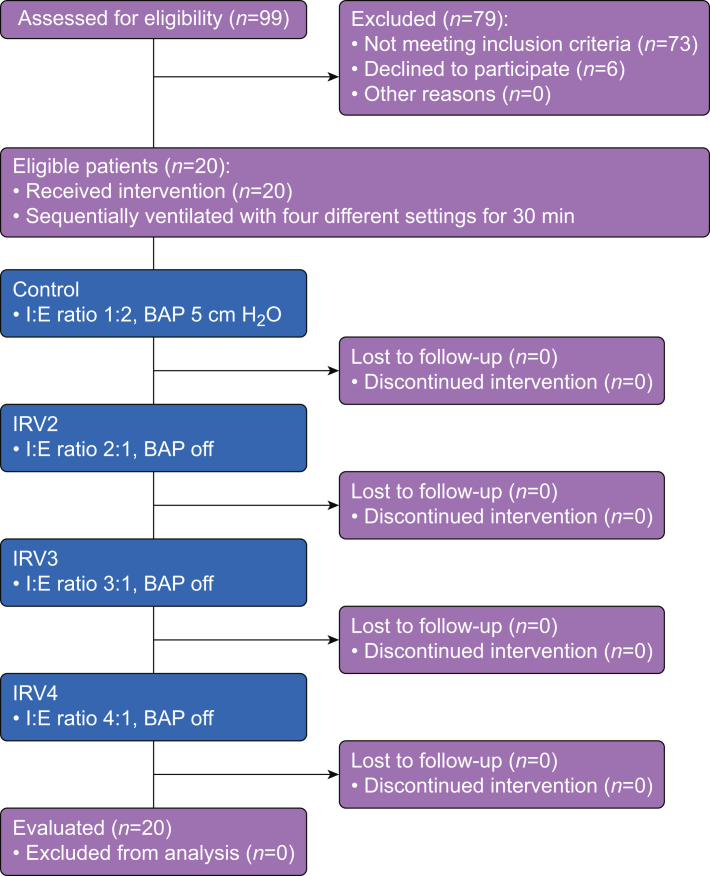
CONSORT diagram for patient inclusion. CONSORT, Consolidated Standards of Reporting Trials; IRV, inverse-ratio ventilation; PC, pressure control; BAP, baseline airway pressure.

Out of the 99 patients screened, 73 patients did not meet the inclusion criteria and six patients refused participation. Thus, 20 patients were included in the analysis. No patient was lost to follow-up (Fig 2). The patient characteristics and respiratory measurements before intervention in the supine position without CO_2_ pneumoperitoneum are summarised in Table 2. The patients' lungs were ventilated with no respiratory difficulties in the supine position without CO_2_ pneumoperitoneum.

**Table 2:** Patient characteristics and respiratory variables before intervention, in the supine position with no CO_2_ pneumoperitoneum (*n*=20). Data are expressed as the median (inter-quartile range [IQR]) or number. FVC, forced vital capacity; FEV1, forced expiratory volume in 1 s; FRC, functional residual capacity; VD_phys_, physiological dead space; VD_shunt_, shunt dead space; VD_aw_, airway dead space; VD_alv_, alveolar dead space.

Variable	Value
*Characteristics*	
Age (yr)	66 (62.3–72)
Height (cm)	165 (160.8–170)
Weight (kg)	76.5 (71–81)
BMI (kg m^−2^)	27.5 (25.3–30.1)
FVC %predicted (%)	97 (93–110)
FEV1 %predicted (%)	82 (77.8–86)
ASA physical status 1/2/3 (*n*)	3/17/0
*Ventilatory settings*	
Mode	Volume-targeted pressure control
Target tidal volume (ml)	500 (494–500)
Target tidal volume per kg body weight (ml kg^−1^)	6.49 (6.14–7.09)
Ventilatory frequency (bpm)	12
Inspiratory:expiratory (ratio)	1:2
Baseline airway pressure (cm H_2_O)	5
*Respiratory parameters*	
Plateau pressure (cm H_2_O)	15 (14–17)
Static compliance (ml cm H_2_O^−1^)	46.2 (39.3–50.5)
FRC (ml)	1861 (1597–2129)
*P*ao_2_/FIO_2_ (ratio)	322 (262–322)
*P*aco_2_ (kPa)	4.8 (4.5–5.1)
*Dead space (VD*_*phys*_*=VD*_*aw*_*+VD*_*alv*_*+VD*_*shunt*_*)*	
VD_phys_ (ml)	161.6 (146.1–179.2)
VD_shunt_ (ml)	39.7 (23.9–48.1)
VD_aw_ (ml)	89.2 (69.9–96)
VD_alv_ (ml)	39.1 (36.5–45.3)

### Effect of IRV

The ventilator settings and respiratory and haemodynamic variables during intervention under the Trendelenburg position and CO_2_ pneumoperitoneum are shown in Table 3. The ventilatory frequency and target tidal volume did not change throughout the study in any patient. Static compliance was low, and there were no significant differences between the groups. Because of the low static compliance, the target tidal volume per kg body weight was 6.37 (5.97–6.64) ml kg^−1^, with the plateau pressures' upper limit being 30 cm H_2_O. FRC and auto-PEEP increased significantly as the I:E ratio increased. For dead space, VD_phys_, VD_shunt_, and VD_aw_ were significantly lower in all three IRVs than in the control; however, there were no significant differences among IRV2, IRV3, and IRV4. IRV resulted in lower cardiac index and higher SVV than the control.

**Table 3:** Intraoperative ventilator settings and respiratory and haemodynamic variables during Trendelenburg position and CO_2_ pneumoperitoneum. Data are expressed as the median (inter-quartile range [IQR]). *F*- and *P*-values are obtained using the Friedman test. ^§^*P*<0.05, *vs* control; ^†^*P*<0.05 *vs* IRV2; ^‡^*P*<0.05 *vs* IRV3, from the Wilcoxon signed-rank test including Bonferroni correction. IRV, inverse ratio ventilation; FRC, functional residual capacity; VD_phys_, physiological dead space; VD_shunt_, shunt dead space; VD_aw_, airway dead space; VD_alv_, alveolar dead space; NA, not available.

Variable	Control	IRV2	IRV3	IRV4	F Value	P value
*Ventilatory settings*						
Mode	Volume-targeted pressure control					
Target tidal volume (ml)	475 (450–500)	475 (450–500)	475 (450–500)	475 (450–500)	NA	NA
Target tidal volume per kg body weight (ml kg^−1^)	6.37 (5.97–6.64)	6.37 (5.97–6.64)	6.37 (5.97–6.64)	6.37 (5.97–6.64)	NA	NA
Ventilatory frequency (bpm)	12	12	12	12	NA	NA
Inspiratory:expiratory ratio	1:2	2:1	3:1	4:1	NA	NA
Baseline airway pressure (cm H_2_O)	5	0	0	0	NA	NA
*Respiratory parameters*						
Inspired tidal volume (ml)	452 (433–460)	446 (426–467)	444 (425–466)	442 (419–462)	1.14	0.767
Expired tidal volume (ml)	438 (421–448)	441 (430–470)^§^	440 (427–468)^§^	437 (425–466)^§^	11.58	0.009
Plateau pressure (cm H_2_O)	26 (24–27.8)	22 (20.3–25.8)^§^	22.5 (22–25)^§^	24 (22–25)^§†‡^	43.17	<0.001
Mean airway pressure (cm H_2_O)	11.5 (11–12.75)	14 (13–16.75)^§^	16 (15–19)^§†^	19 (17–20)^§†‡^	57.68	<0.001
PEEP (cm H_2_O)	5 (5–5)	0 (0–0)^§^	0 (0–0)^§^	0.5 (0–1)^§†‡^	53.46	<0.001
Auto-PEEP (cm H_2_O)	0 (0–1)	1 (1–2)^§^	2.5 (2–3)^§†^	3.5 (3–5)^§†‡^	53.68	<0.001
Total PEEP (cm H_2_O)	5 (5–6)	1 (1–2)^§^	2.5 (2–3)^§†^	4 (3–5.8)^†‡^	47.66	<0.001
Static compliance (ml cm H_2_O^−1^)	21.9 (19.1–24.7)	21.6 (19.4–23.6)	22.7 (19.7–25.1)	23.1 (19.9–26.6)	6.06	0.109
FRC (ml)	1149 (898–1386)	1485 (1018–1717)^§^	1602 (1209–1775)^§†^	1757 (1337–1955)^§†‡^	47.7	<0.001
*P*ao_2_/F_I_o_2_ (ratio)	337 (234–413)	298 (236–418)	300 (241–423)	322 (259–431)^†‡^	12.45	0.006
*P*aco_2_ (kPa)	5.4 (4.8–5.6)	5.3 (4.7–5.5)^§^	5.2 (4.5–5.3)^§^	5 (4.4–5.4)^§†‡^	24.86	<0.001
Dead space (VD_phys_=VD_aw_+VD_alv_+VD_shunt_)						
VD_phys_ (ml)	139.4 (123–151.5)	111.6 (88.9–134.1)^§^	104.2 (86.3–124.3)^§^	96.7 (84.3–124)^§^	38.58	<0.001
VD_aw_ (ml)	59.3 (52.6–76.4)	51.6 (43.3–62.5)^§^	56.2 (46.2–58.8)^§^	54.1 (38.3–62.4)^§^	21.21	<0.001
VD_alv_ (ml)	46.3 (39.5–55.6)	46.6 (43.3–54.6)	50.1 (42–55.3)	48.3 (42.7–58.9)	5.82	0.121
VD_shunt_ (ml)	26.6 (15.6–44)	4.4 (–9.9 to 28.8)^§^	4.7 (–8.9 to 13.4)^§^	–2.3 (–12.3 to 12)^§^	33.66	<0.001
*Haemodynamic variables*						
Mean arterial blood pressure (kPa)	13.1 (12–14.2)	11.8 (11–12.9)^§^	11.4 (10.4–13.2)^§^	11.1 (10.3–12.2)^§†^	33.81	<0.001
Heart rate (beats min^−1^)	62 (56.5–73.5)	60 (56.5–71.8)	60 (57–68.8)^§^	59.5 (57.3–66.8)	11.54	0.009
Cardiac index (L min^−1^ m^−2^)	2.8 (2.38–3.35)	2.8 (2.3–3.55)	2.45 (2–3.25)^†^	2.1 (1.75–3.1)^§†^	20.98	<0.001
Stroke volume variation (%)	7.5 (6–9.8)	11 (8–16.8)^§^	12 (10–13)^§^	12 (9–15.8)^§^	38.39	<0.001

### Factors affecting the FRC

**Fig. 3:**
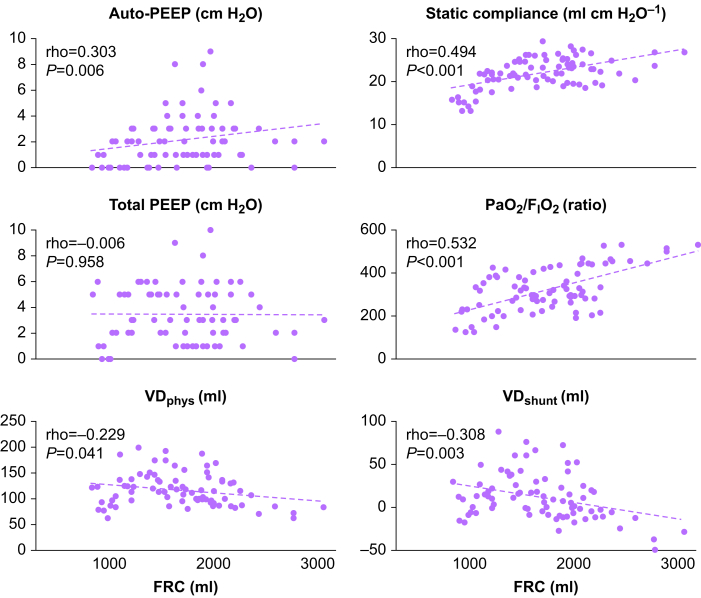
Correlation of FRC with other variables, auto-PEEP, total PEEP, static compliance, *P*ao_2_/F_I_o_2_, VD_phys_, and VD_shunt_. Spearman's rank correlation is used to assess the relationship between the variables. FRC, functional residual capacity; VD_phys_, physiological dead space; VD_shunt_, shunt dead space.

Multiple regression analysis of the whole data set showed that BMI (*P*=0.002), I:E ratio (*P*=0.001), and target tidal volume (*P*<0.001) were independently associated with FRC (adjusted *R*^2^=0.337, *P*<0.001). However, other factors, including %predicted FVC, %predicted FEV1, or BAP, were not. Spearman's rank correlation showed that FRC values were correlated with auto-PEEP, static compliance, *P*ao_2_/F_I_O_2_, VD_shunt_, and VD_phys_, but not with total PEEP (Fig 3).

### Complications

There were no respiratory complications during the perioperative period with auto-PEEP, peripheral oxygen saturation, and E_T_CO_2_ maintained within the predetermined limits in all patients. However, in one patient (a smoker with FEV_1_ of 79%), auto-PEEP reached 9 cm H_2_O during the IRV4 settings with FRC of 1752 ml compared with 1157 ml at the control settings.

## Discussion

The effect of an inverse I:E ratio on FRC during pneumoperitoneum remains unclear. We found that the larger the I:E ratio with vtPC-IRV, the higher was the FRC. Furthermore, the I:E ratio – but not BAP – was an independent predictor of FRC. FRC was correlated with auto-PEEP but not with PEEP or total PEEP.

Tan and colleagues^16^ reported that FRC was correlated with auto-PEEP during mechanical ventilation with a normal I:E ratio in patients with an exacerbation of chronic obstructive pulmonary disease. In contrast, auto-PEEP was generated by a short expiratory time with inverse I:E ratio in our study. Futier and colleagues^17^ reported that a high BAP of 10 cm H_2_O with an I:E ratio of 1:2 effectively improved FRC and respiratory mechanics in non-obese patients. However, the effects of high BAP were diminished in patients with obesity during general anaesthesia with neuromuscular blocking agents.^17^ In a pig model of intra-abdominal hypertension (low static compliance model), BAP of up to 15 cm H_2_O did not prevent FRC decreases.^18^ Nestler and colleagues^19^ reported that in individualised BAP titrated using electrical impedance tomography, a mean PEEP value of 18 cm H_2_O increased end-expiratory lung volume and improved *P*ao_2_/F_I_O_2_ in obese patients during elective laparoscopic surgery.

In our study, patients with obesity must have had low static compliance during CO_2_ pneumoperitoneum in the Trendelenburg position. Therefore, it was difficult to increase FRC because of the low levels of BAP with a long expiratory time (I:E ratio of 1:2), in the same way as previously reported. Thus, our results suggest that short expiratory time with IRV accompanied by increased auto-PEEP would effectively increase FRC under such conditions with a low static compliance. However, although increased FRC may prevent atelectasis, it has a potential risk of hyperinflation of the normal alveoli and circulatory compromise.

Atelectasis may obstruct alveolar expansion, inducing relative hyperperfusion (V̇_A_/Q̇<1) in the impaired alveolar unit and may redistribute the inflated gas toward the healthy alveolar unit excessively, causing relative hyperinflation (V̇_A_/Q̇>1). Similar to lung recruitment manoeuvres, PC-IRV can decrease pulmonary blood flow to the ventilated alveoli, inducing relative hyperinflation (V̇_A_/Q̇>1). Moreover, it is likely to redistribute the pulmonary blood flow toward the non-ventilated alveoli, promoting relative hyperperfusion (V̇_A_/Q̇<1).

Our analysis of dead space helped detect the heterogeneous distribution of relative hyperinflation and hyperperfusion presenting as increased VD_alv_ and increased VD_shunt_, respectively. Thus, VD_alv_ and VD_shunt_ improve significantly when the open-lung approach ventilation strategy improves the heterogeneous distribution of alveolar units with relative hyperinflation and hyperperfusion, resulting in minimised VD_phys_.^10^^,^^12^^,^^19^, ^20^, ^21^

The reduction of VD_phys_ during vtPC-IRV results from decreases in VD_shunt_ and VD_aw_ accompanied by unchanged VD_alv_. The prolonged plateau time likely afforded sufficient inflation of alveoli with compromised expansion. In addition, the short expiratory time with increased auto-PEEP led to an increased FRC, and both resulted in decreased VD_shunt_. vtPC-IRV slightly reduced VD_aw_, suggesting that prolonged plateau time enhanced diffusion of gas within the airway space. A prolonged plateau time may enhance gas diffusion from the pulmonary artery to the alveoli. Excessive plateau pressure or circulatory suppression might increase intra-alveolar V̇_A_/Q̇>1 mismatch. Thus, PC-IRV can potentially increase VD_alv_ under high-plateau pressure conditions with circulatory suppression. In this study, vtPC-IRV induced significant decreases in the mean arterial blood pressure and cardiac index, with increased SVV. However, the SVV and cardiac index were controlled within the normal range. No significant change in VD_alv_ by vtPC-IRV indicated successful management of vtPC-IRV with moderate plateau pressure and circulatory dynamics.

VD_phys_ in all IRV settings were significantly lower than those in the control setting, suggesting that vtPC-IRV would be an open-lung approach ventilation strategy suitable for a low static compliance situation. However, there were no significant differences in VD_phys_ or VD_shunts_ among IRV2, IRV3, and IRV4, which indicates that with respect to dead space, an I:E ratio of 3:1–4:1 has a smaller benefit than an I:E ratio of 2:1, although larger I:E ratios induced larger increases in FRC.

Earlier studies showed that the higher the BAP, the higher was the FRC^21^^,^^22^; however, a high BAP of 14–20 cm H_2_O increased dead space under normal static compliance.^14^^,^^23^^,^^24^ Fengmei and colleagues^25^ concluded that the optimal BAP was 12 cm H_2_O because it induced the highest compliance in conjunction with the lowest dead space, indicating a maximum amount of effectively expanded alveoli. These studies were usually conducted at a high BAP, with an I:E ratio of 1:2. A long expiratory time (I:E ratio of 1:2) may contribute to reduced FRC and induce atelectasis. Furthermore, increasing FRC by BAP under long expiration time (I:E ratio of 1:2) with low respiratory compliance is difficult. IRV with a short expiratory time and high auto-PEEP even with low respiratory compliance effectively increases FRC. However, IRV is not commonly used or safe. It needs an anaesthesia ventilator equipped with an expiratory flow-time wave monitor to avoid lung hyperinflation, adequate haemodynamic management, moderate muscle relaxation, and the expertise of an anaesthesiologist. Therefore, IRV with an I:E ratio of 3:1–4:1 is not recommended. However, a short expiratory time with IRV would enhance the BAP effect of increasing FRC in situations with low static compliance. We believe that vtPC-IRV with an I:E ratio of 1:1–2:1 and a BAP of 5–7 cm H_2_O is a safe and practical open-lung approach ventilation strategy in situations with low static compliance, which may provide sufficient total PEEP and appropriate inflation of the slow opening alveolar components.

## Limitations

In this study, the ventilator settings were sequentially changed at intervals of 30 min, and the measurements were obtained at the end of each setting. As the sequence was not randomised, the results might be influenced by time-dependent changes or trends in respiratory status during the 2-h intervention period. We cannot rule out this possibility. However,^12^ VD_phys_ and VD_shunt_ increased within 2 h intraoperatively, and atelectasis probably developed in ventilator conditions with an I:E ratio of 1:2 or 2:1, suggesting that FRC may decrease, and the dead space components may increase with time. Therefore, it is conceivable that our findings of IRV acutely increasing FRC and decreasing VD_phys_ and VD_shunt_ were attributable to ventilator settings, rather than time-dependent changes.

Atelectasis likely develops heterogeneously within hours; however, we only measured acute changes in the respiratory and circulatory status in each 30-min period of ventilator setting. Changes over a longer period or the actual degree of atelectasis were not evaluated. Further studies evaluating the development of atelectasis using electrical impedance tomography, a useful tool for evaluating the heterogeneous development of atelectasis over time,^26^^,^^27^ are warranted to establish a safe and practical open-lung approach ventilation strategy.

## Conclusions

vtPC-IRV with short expiratory time and high auto-PEEP effectively increases FRC during robot-assisted laparoscopic radical prostatectomy in patients with obesity. The higher FRC is observed with larger I:E ratio in vtPC-IRV. Furthermore, vtPC-IRV decreases the shunt and physiological dead space, indicating that vtPC-IRV can be used as an open-lung approach ventilation strategy in cases of low respiratory compliance. However, an I:E ratio of more than 2:1 does not improve dead space further, suggesting that using an I:E ratio of more than 2:1 is not required under these conditions.

## Authors' contributions

Study design: GH, TA.

Data acquisition: GH, YY, HO, MS, KM.

Data analysis: GH.

Drafting of the article: GH, TA.

All authors interpreted the data and approved the final version of the manuscript.

## Funding

The authors received no specific funding for this work.

## Declarations of interest

The authors declare that they have no conflicts of interest.
