# Supplementary material for: Effects of volume-targeted pressure-controlled inverse ratio ventilation on functional residual capacity and dead space in obese patients undergoing robot-assisted laparoscopic radical prostatectomy
Source: BJA Open. 2022 Jul 20;3:100020. doi: 10.1016/j.bjao.2022.100020 (PMC10430846; doi:10.1016/j.bjao.2022.100020)
Supplement: Multimedia component 2 [file mmc2.docx]

**Supplementary data**

**1. Components of dead space and volumetric capnography**

In traditional volumetric capnography, the expired CO_2_ fraction on the Y axis is plotted against the V_TE_ on the X axis. This analysis has been called the single breath test-CO_2_ (SBT-CO_2_). Normally, the expirogram is divided into three phases. Phase I represents CO_2_-free and pure dead space, phase II represents the transition between airway and alveolar gas, and phase III represents alveolar gas. Phase II ends at the intersection of the slope of phase II and the slope of phase III. We present a volumetric capnogram with a transposed x-y axis as shown in Fig. 1. In 1948, Fowler applied the equal area method to phase II of the nitrogen expirogram after pure O_2_ inspiration, which he claimed was the geometric representation of Bohr’s formula.^1^ Thereafter, this dividing line was called the ‘Fowler Line’. In 1981, Fletcher and colleagues^2^ presented a review and theoretical analysis of dead space by SBT-CO_2_, and they showed the geometric representation of Enghoff’s modification of Bohr’s formula. They drew a line of F_a_CO_2_ above the CO_2_ expirogram, and the area between this line and the CO_2_ expirogram was defined as physiological dead space (VD_phys_). Subsequently, airway dead space (VD_aw_) was defined by applying Fowler’s equal area method to phase II. Finally, the area between the F_a_CO_2_ line and Phase III of the CO_2_ expirogram was defined as alveolar dead space (VD_alv_). VD_phys_ and its subdivisions of VD_aw_ and VD_alv_ were shown to correspond to each of the VDs on the SBT-CO_2_ expirogram. VD_aw_ separated by the Fowler Line is not a static volume of the respiratory tract, but a dynamic and functional volume affected by various aspects of the flow pattern, such as flow rate, respiratory rate, and I/E ratio.^2^ Fletcher and colleagues defined VD_alv_ as VD_phys_ - VD_aw_ geometrically. Regardless of the name ‘alveolar dead space’, the VD_alv_ was supposed to include the effect of venous admixture.^2^ In 2006, Tang, Turner, and Baker proposed a new equal area method to represent VD_phys_ on the volumetric axis which made it easy to compare with other VDs.^3^ We will call this VD_phys_ dividing line the ‘Tang Line’ (Fig. 1).

参考：Fletcher1981


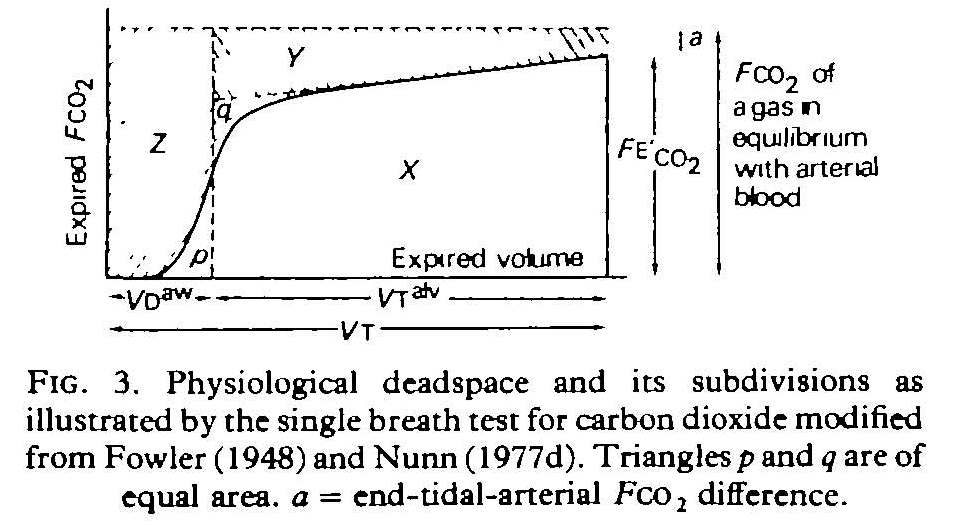


**2. Novel volumetric capnography and VD_resp_**

We used a non-invasive breath-by-breath volumetric capnography monitoring device developed by Senko Medical Instrument Co., Ltd. (Tokyo, Japan) to estimate dead space components based on a novel analytic method. This device uses mainstream PHASEIN IRMA (Masimo Sweden AB); an infrared CO_2_ sensor for CO_2_ measurement; and EZ-Flow (TREYMED, Inc., US), a fixed orifice, differential pressure device for volume measurement. Analogue data output from these sensors are sent to the A/D converter, and the digital data are sent to a PC. Specialised software for novel dead space analysis was developed in Microsoft Visual Studio using the C language. The first unique point of this method is that the X axis represents FCO_2_ and the Y axis represents tidal volume, for geometrical reasons. Second, this method is based on a new theoretical model that can simultaneously determine the respiratory dead space (VD_resp_) and F_A_CO_2_ geometrically in a breath-by-breath manner, as shown in Fig. 2. The volume from the start of expiration to the mixed expired CO_2_ fraction point on the Y axis is defined as VD_resp_. Then, F_A_CO_2_ is defined as VCO_2_･(V_TE_ - VD_resp_)^-1^. Similar to VD_Bohr_, VD_resp_ is a functional evaluation of the difference of the CO_2_ partial pressure between alveolar and mixed expired gas [VD_resp_ = V_TE_･(P_A_CO_2_ - P_E_CO_2_)･P_A_CO_2_^-1^].

**3. New method of dividing phases II and III**

VD_aw_ is analysed by Fowler’s equal area method non-invasively as well as geometrically. The third unique point of this novel volumetric capnography is that the maximum point of multiplication of (x, y) values on the expirogram are used for dividing phases II and III. To determine the VD_aw_ by applying Fowler’s equal area method to the second phase, it is essential to understand how to divide phase II and III properly and stably. The most popular method of determining the dividing line is by applying linear regression to each phase and dividing at the intersection of the two lines. However, this method has the disadvantage of using linear regression without determination of the ranges of the two phases. As a new method that can eliminate this arbitrariness, we focused on the changes in the inner rectangular area that is inscribed in one of the corners to a point (x, y) on the expirogram. This area increases initially, reaches the maximum at a certain point (x, y), and subsequently decreases. Because both phases normally have different inclinations, such changes are necessary. Therefore, this method of dividing phases II and III at the maximum point of multiplication of (x, y) values on the expirogram is considered to be reasonable (Fig. 1).

**References**

1. Fowler WS. Lung function studies; the respiratory dead space. *Am J Physiol* 1948; **154**:405–416
2. Fletcher R, Jonson B, Cumming G, et al. The concept of deadspace with special reference to the single breath test for carbon dioxide. *Br J Anaesth* 1981; **53**:77–88.
3. Tang Y, Turner MJ, Baker AB. A new equal area method to calculate and represent physiologic, anatomical, and alveolar dead spaces. *Anaesthesiology* 2006; **104**:696–700.

**Fig.1. Overall schema of the new volumetric capnography**

VD_resp_ is a geometric representation of Bohr’s formula. Phase II is divided from phase III at the maximum point of multiplication of (x, y) values on the expirogram. VD_aw_ is defined by the ‘Fowler Line’, and as a natural result, VD_alv_ = VD_resp_ - VD_aw_. VD_phys_ is defined by the ‘Tang Line’, and we defined [VD_phys_ - VD_resp_] as VD_shunt_. VCO_2_ = V_TE_ × FECO_2_ = V_A_ × F_A_CO_2_ = V_a_ × F_a_CO_2_. Each multiplication can be expressed as the square area consisting of the segments corresponding to these CO_2_ fractions and volumes, and these three square areas are equal to VCO_2_. VD_resp_, respiratory dead space; VD_aw_, airway dead space; VD_alv_, alveolar dead space; VD_phys_, physiological dead space; VD_shunt_, shunt dead space; V_A_, alveolar tidal volume (virtual); V_a_, arterial tidal volume.


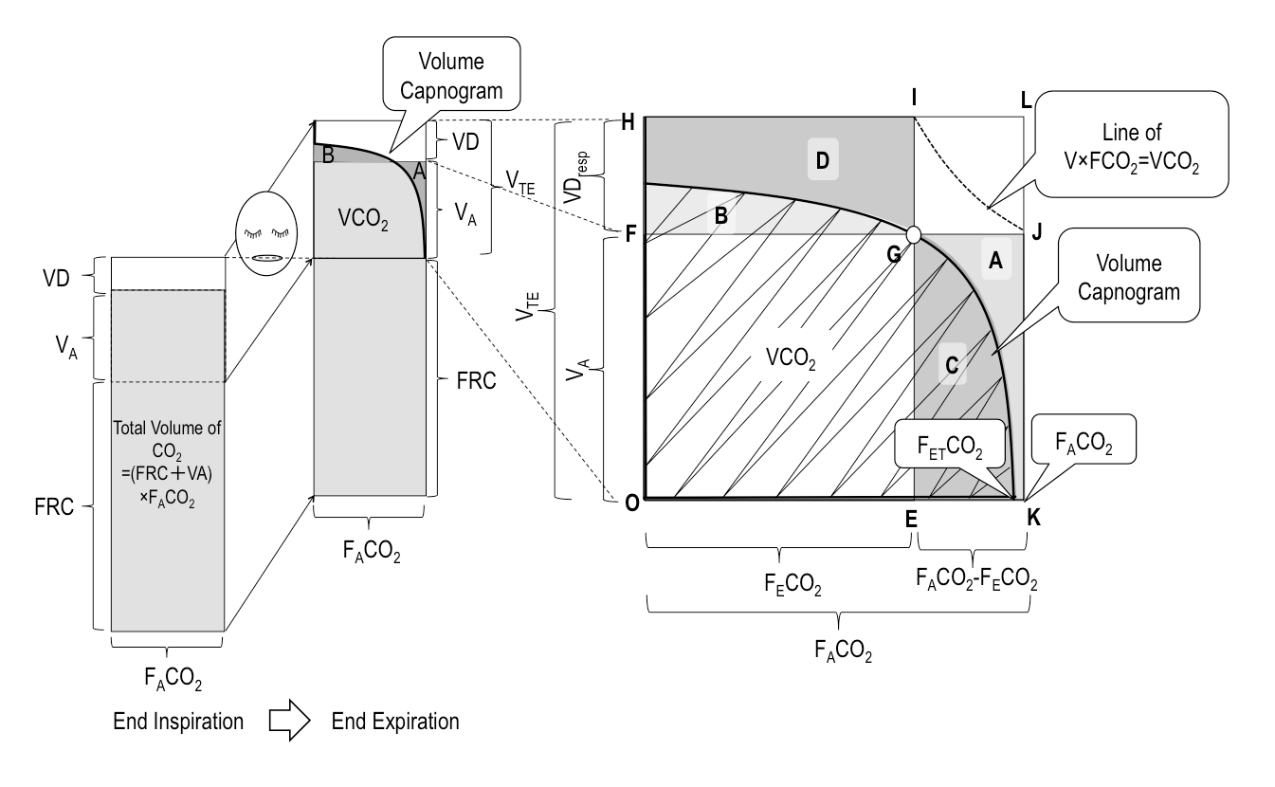


**Fig.2. New theoretical model and geometric representation of Bohr’s formula [V_TE_ × F_E_CO_2_ =V_A_ × F_A_CO_2_].**

In volume capnography, VCO_2_ is presented as the area confined by the X axis, the Y axis, and the volume capnogram, corresponding to the area B plus C in the figure. VCO_2_ can be also calculated by V_TE_ × F_E_CO_2_, corresponding to the area B plus D. Alternatively, it can be calculated by V_A_ × F_A_CO_2_, corresponding to the area defined by the points F-O-K-J. Because G is a mixed expired fraction point, the area C is equal to the area D. When we define H-F as VD_resp_, F_A_CO_2_ also can be defined at the same time. G is the single point on the volume-capnogram at which ‘A = B’ and ‘C = D’ can be achieved. In this figure, Bohr’s VD/V_TE_ ratio = VD_resp_/V_TE_ = (F_A_CO_2_ - F_E_CO_2_) F_A_CO_2_^-1^. This equation tells us that the VD/V_TE_ ratio is a projection of the fractional relationship on the X axis to the volumetric relationship on the Y axis. The inverse proportion line of [Volume × FCO_2_ = VCO_2_] makes this possible. V_TE_, expired tidal volume; VCO_2_, expired tidal volume of CO_2_; VD_resp_, respiratory dead space; V_A_, alveolar tidal volume. FCO_2_, fraction of CO_2_ partial pressure [FCO_2_ = PCO_2_ (Pb-PH_2_O)^-1^ = PCO_2_ (760-47)^-1^, 1 atm BTPS condition]; P_E_CO_2_, mixed expired partial pressure of CO_2_; P_ET_CO_2_, end tidal partial pressure of CO_2_. P_A_CO_2_, alveolar partial pressure of CO_2_, and P_a_CO_2_, arterial partial pressure of CO_2_, are expressed as FCO_2_ in this figure.
